# Detecting urban commercial patterns using a latent semantic information model: A case study of spatial-temporal evolution in Guangzhou, China

**DOI:** 10.1371/journal.pone.0202162

**Published:** 2018-08-20

**Authors:** Shili Chen, Haiyan Tao, Xuliang Li, Li Zhuo

**Affiliations:** 1 School of Geography and Planning, Sun Yat-sen University, Guangzhou, Guangdong, China; 2 Urbanization Institute of Sun Yat-sen University, Sun Yat-sen University, Guangzhou, Guangdong, China; 3 Guangdong Provincial Key Laboratory for Urbanization and Geo-simulation, Sun Yat-sen University, Guangzhou, Guangdong, China; 4 Center of Integrated Geographic Information Analysis, School of Geography and Planning, Sun Yat-sen University, Guangzhou, Guangdong, China; National University of Singapore, SINGAPORE

## Abstract

With rapid economic growth since the 21st century, cities in China have experienced considerable economic and social reconstruction. Driven by rapid industrialization, urban spatial structures are undergoing evolution and change. Therefore, this paper analyzes the processes and mechanisms associated with the evolution of the commercial spatial structure in Guangzhou after the financial crisis in 2008 based on both theoretical and empirical analyses. We use a Dirichlet multinomial regression (DMR) model to extract latent semantic information and determine urban functional areas from global positioning system (GPS) and point-of-interest (POI) data collected in Guangzhou in 2009 and 2013. In addition, we use movement patterns and POI data to identify the evolution of Guangzhou's commercial zones from 2009 to 2013. The results show that the urban commercial structure in Guangzhou gradually changed from a single-center model to a multi-center model with dispersed clusters and that the distribution of the entire spatial structure changed. Meanwhile, Guangzhou’s commercial structure not only varied over time but also exhibited specific geographical features. This paper demonstrates that the proposed method can clearly identify the boundary of the commercial area in Guangzhou and provides a valid spatial-temporal model of change in the city. Moreover, this study not only expounds the future development trends of the urban spatial structure in Guangzhou from a microcosmic perspective but also provides a scientific basis for clarifying the spatial locations and development advantages of urban functions within the city.

## Introduction

We travel from our residences to our workplaces every weekday. If our workplaces are close to our residences, then we need to travel only a short time and distance to reach our destination. However, if our workplaces are far away, we must use transportation. Therefore, urban traffic peaks occur during the morning and evening, producing a pendulum traffic pattern. Additionally, as a result of pendulum traffic, some regions have a much larger population during the day than during the night, a phenomenon known as the “hollowing out” of a city center.

This phenomenon occurs as urbanization increases, the urban population surges, and urban built-up areas expand, causing a change in the urban spatial structure. According to statistics, the urban population currently accounts for over 50% of the total population and will likely reach 69.6% by 2050 [1]. The global built-up area is also continually expanding and changing the spatial structure of the urban interior from the concentric circle structure [2], fan-shaped structure [3], and multi-core structure [4] models proposed by the Chicago School to the multiple centers and multi-center networks [5,6] proposed by the Los Angeles School. Such changes in urban spaces are based on changes in the spatial distribution of urban commerce.

Urban businesses serve as the main functions of cities and emerge with the establishment of cities [7]. The commercial space is one of the most active areas in cities and has a significant impact on urban development and the evolution of the urban spatial structure. Therefore, research in this area constitutes an important part of urban geography studies. As business contacts between countries and regions strengthen and as more distribution centers and exchange markets emerge, research on commercial structures is improving, especially with the application of a series of scientific theories and methods, such as quantitative geography, behavioral geography and operational research approaches.

Previous studies of commercial space have mainly focused on two research areas involving the intra-urban and spatial structures of urban systems: the commercial carrier and the commercial subject. Most studies of commercial carriers have employed rent theory [8], central place theory [9–11], measurement methods [12–14], etc. to research the commercial space structure. In studies of business subjects, some scholars have proposed research models of consumer behaviors, business circles, etc. [15–25], while others have suggested more diverse models to describe urban commercial space from the perspective of the market structure and size of the urban center [13,24]. Some recent studies have introduced specific hypotheses to create more dynamic and realistic models, especially considering consumer factors [15,16,19] and the changes in commercial center systems based on central place theory [17,18,20,21,23,25]. However, almost all the data used in these studies were derived from traditional surveys, which are expensive and require considerable time and effort. Due to data limitations, these studies did not analyze the structure and evolution of commercial spaces over any given period.

In recent years, the development of information and telecommunication technology and the accessibility to big data have given urban geology a new perspective [26–28], including new research methods to study urban spatial structures. Big data has been widely applied to study urban spatial structures. Such studies have analyzed urban spatial structures and urban functional zoning based on mobile data [29,30], bus data [31–33], GPS data [34–38], and location-based service (LBS) data [39–43]. Additionally, a city’s taxi data reflect the travel needs of residents and have low costs, high accuracy, wide coverage, and immediate availability. Compared to traditional research on urban spatial structures, studies that identify a city’s spatial structure through GPS data not only require less time for field research but also avoid the need for survey results. Moreover, the results of these studies are more accurate due to the large sample size. Some scholars have previously used big data to study people’s travel behaviors [40,44–46]. In addition, some researchers have used taxi data to analyze several aspects of land use. For example, Pan used taxi GPS traces to perform land-use classification and found that taxi pick-up/set-down patterns corresponded to the land-use classes of regions [47]. Yang used GPS data to analyze land-use patterns in Washington, DC, and found that taxi movement were strongly associated with land-use patterns [48]. In addition, Nong used taxi data to delimit the boundaries of retail centers and determine their hierarchical characteristics [49]. However, few scholars have employed big data to analyze customer behaviors and quantitatively evaluate commercial spatial structures. Furthermore, Chinese studies in this research area are very rare. From the perspective of economics, customer behavior plays a significant role in the formation and evolution of commercial structures. The travel behaviors of taxis in a city reflect the customer behaviors of residents during their activities.

Based on floating car data from 2009 and 2013, this paper takes Guangzhou as an example and identifies its commercial structure after the financial crisis using a latent semantic analysis. Specifically, the study analyzes the mobility patterns of Guangzhou residents between different regions and examines point-of-interest (POI) data on related factors that compose a commercial zone, which are used to identify Guangzhou’s commercial structure. The results reveal that the commercial structure of Guangzhou transformed from a single center to a decentralized, polycentric network between 2009 and 2013, and the distribution of the overall spatial structure underwent considerable change. Meanwhile, the commercial structure of Guangzhou not only changed over time but also displayed unique regional characteristics. The results prove that the adopted method can be used to effectively identify commercial areas in Guangzhou and to investigate the evolution of the commercial spatial structure of this city after the financial crisis.

## Study area and data

### Study area

**Fig 1:**
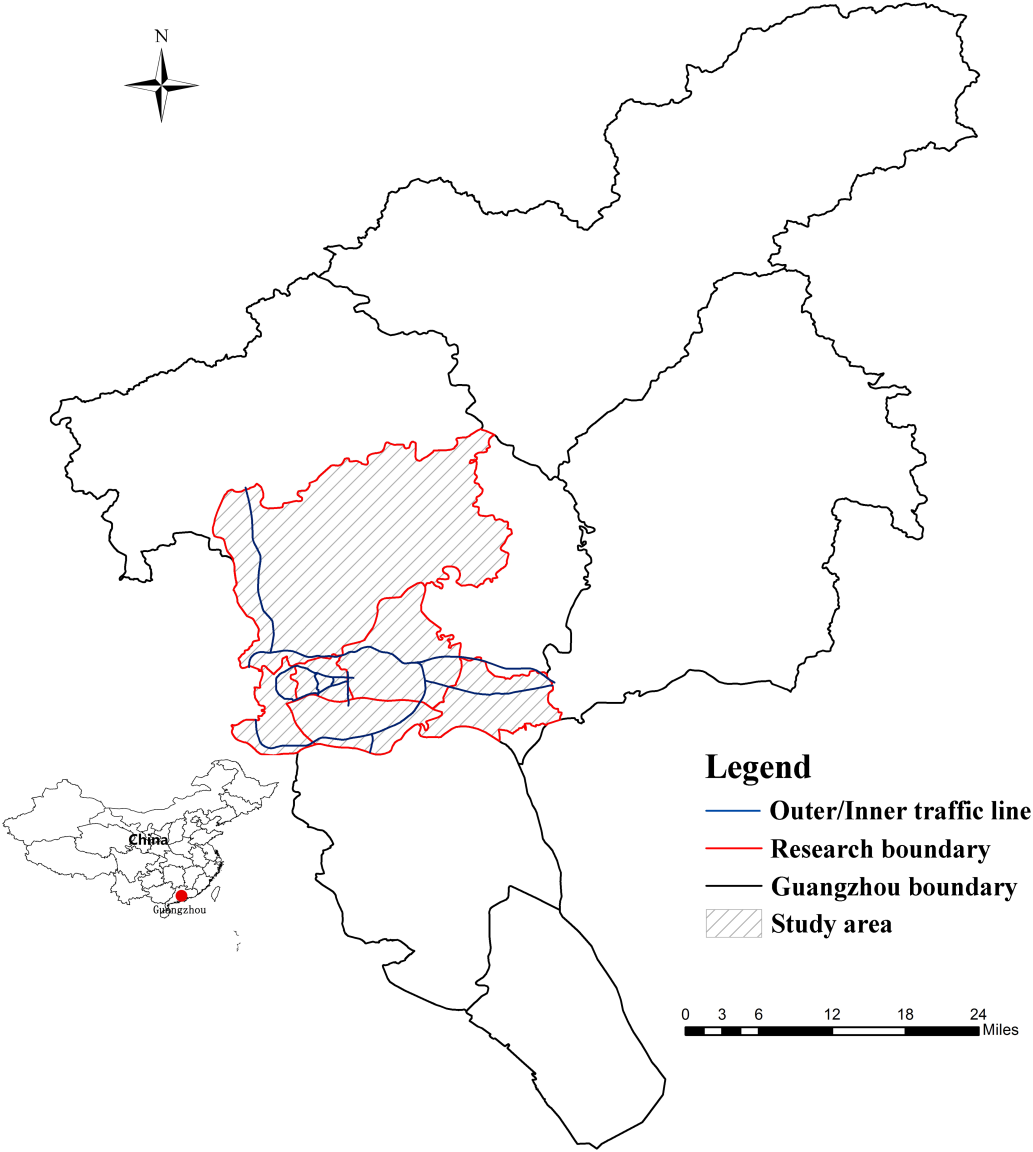
Study area. The figure shows the location of Guangzhou in China and the study area in Guangzhou.

The research area of this paper is the downtown area of Guangzhou in China (longitude 112° 57´~114° 03´ and latitude 22° 26´~23° 56´). To ensure the representativeness of the sample, we consider both the spatial distribution and temporal evolution of the research objects and select “eight old districts”. Thus, the traditionally defined downtown area before the administrative division adjustment in 2014 is chosen as the research area (among those eight districts, Fangcun District has been incorporated into Liwan District, and Dongshan District has been incorporated into Yuexiu District). Additionally, we divide the study area of Guangzhou into 439 zones based on the distribution of freeways, city expressways, national highways, provincial highways, urban main roads, and secondary main roads (as displayed in Fig 1). However, because the Baiyun district along the upper part of the Huanan Expressway is mainly composed of tourist attractions, farmlands, and mountains, this zone is excluded from the research area.

### Data

China's public transportation system collects passengers’ pickup information but does not record the passengers’ drop-off information. Thus, it lacks complete origin-destination information and cannot provide accurate travel information [50,51]. In addition, due to the fixed ranges of subways and buses, some scholars have shown that the active range of conventional public transport is only 400 meters [52]. The range of a subway station service is generally a 500-meter circular area centered at the station [53], which fails to achieve full coverage of a city. Compared to these types of traffic data, taxi data include pickup and drop-off information and cover an entire city; thus, they are more suitable for exhaustive studies of resident travel.

Meanwhile, many scholars have shown that floating car data can accurately represent road traffic scenarios and human behaviors [54,55]. In Guangzhou, the taxi industry also plays an important role. In terms of the number of public transport passengers, 196 million daily passenger taxi trips were recorded in 2009, accounting for 18.67% of all public transport. By October 2013, the average daily passenger volume of taxis reached 2.2 million, accounting for 14.16% of all public transport. Therefore, floating car data can be used to represent the travel needs of urban residents. To illustrate city expansion and changes in land use and spatial structure, the present study utilizes GPS trajectory data from taxis and POI data that describe city buildings. The floating car data include GPS data from May 11, 2009 (Monday), to May 17, 2009 (Sunday), and from October 8, 2013 (Tuesday), to October 14, 2013 (Monday). The GPS data were collected at an interval of five seconds and included basic information such as the plate number of the taxi, time, longitude, latitude, speed, position, occupancy status, etc. Table 1 shows a sample of taxi data from 2009.

**Table 1:** A sample of taxi data.

License Plate	GPS Time	Longitude	Latitude	Speed	Direction	Car State
Yue A000S9	2009/5/11 0:00	113.2825	23.14107	19	360	4
Yue A000S9	2009/5/11 0:01	113.2827	23.14199	14	28	4
Yue A000S9	2009/5/11 0:02	113.2865	23.14364	21	42	4
Yue A000S9	2009/5/11 0:03	113.2903	23.14471	37	67	4
Yue A000S9	2009/5/11 0:04	113.2917	23.14177	18	156	4
Yue A000S9	2009/5/11 0:05	113.2879	23.13731	24	159	4
Yue A000S9	2009/5/11 0:06	113.284	23.1344	27	258	4
Yue A000S9	2009/5/11 0:07	113.2831	23.1351	39	344	4
Yue A000S9	2009/5/11 0:08	113.2821	23.14132	0	74	4
Yue A000S9	2009/5/11 0:08	113.2826	23.14195	21	352	5

POIs are an essential data type used in navigation, smart transportation, and other LBSs. The POI data in this paper can be divided into 15 categories and 65 types. Each POI is numbered based on the “category name + type name + serial number”, including information such as ID, type, longitude, and latitude. Table 2 shows some sample POI data. According to the objectives and requirements of this study, we combine some of the POI data and divide the data into 29 categories. It is worth noting that this paper does not consider the impacts of different classes of POIs on the results.

**Table 2:** Sample POI data.

ID	Type	RDID	Longitude	Latitude
20000001	81406024	20131990	113.604	22.6478
20000002	81102065	20123966	113.608	22.6464
20000003	81402034	20131990	113.604	22.6474
20000004	80601054	20131990	113.604	22.6473
20000005	81506044	20156064	113.566	22.6554
20000006	80101035	20119062	113.593	22.6292
20000007	81004035	20046330	113.575	22.6407
20000008	81406005	20021084	113.609	22.6431
20000010	80101062	20046302	113.606	22.6449
20000011	81201015	20046305	113.605	22.644
20000012	80701054	20150230	113.623	22.6288
20000013	81102005	20046311	113.607	22.6589
20000018	80603045	20021131	113.607	22.6447
20000019	81001005	20000251	113.568	22.6531

## Methodology

The Dirichlet multinomial regression (DMR) model is a topic model that was proposed by Blei in 2003 [56]. As a widely recognized format for text processing, it can build a model to determine the hidden topics in documents. This approach not only improves upon traditional methods of text similarity calculation but also caters to searches of semantic topics based on large corpora or even massive internet data [56–58]. The model can determine the probability of multiple topics in each document from a corpus and can fully extract the semantic information from a word or sentence. Thus, we use DMR model to analysis the GPS data.

According to the DMR model, each document in the corpus can be regarded as a combination of multiple topics. Each word in the document is associated with a topic. Therefore, if all the words in a document are available, the distribution of topics can be obtained through mathematical derivation.

**Fig 2:**
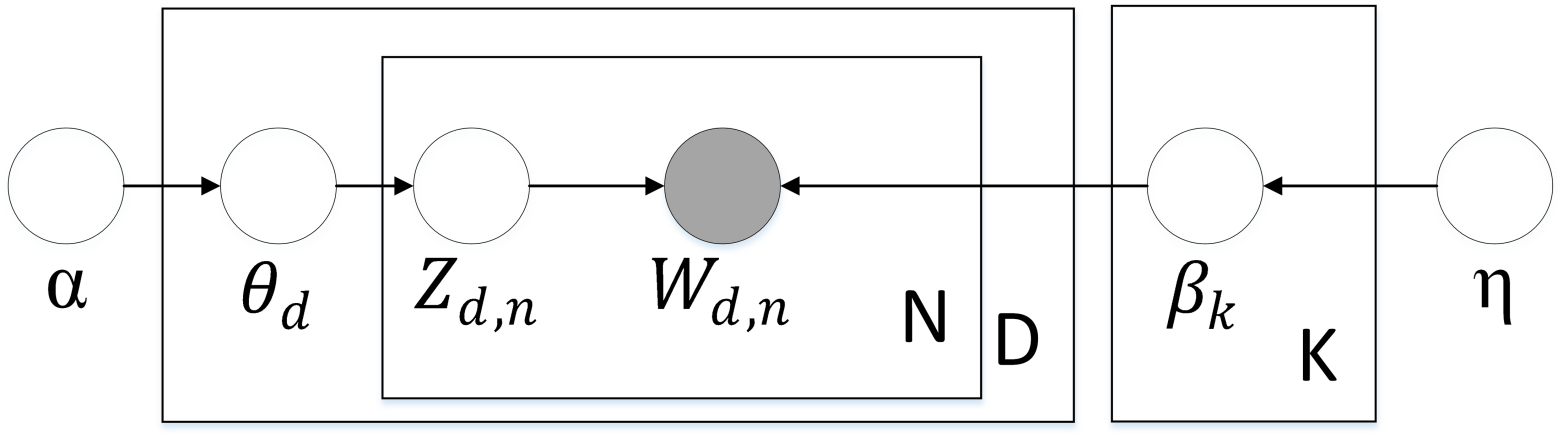
DMR model.

Urban functional zoning and text representation studies are similar to a certain extent. If we regard a region as a document and a function as a topic, then every trip in a region can be considered a word, and a functional zone can be characterized by its agglomeration of activities, intraregional transport infrastructure, resident mobility, and inputs within its borders of interaction [58]. The DMR model [45] is displayed in Fig 2.

Nodes represent random variables and are denoted using different colors and shapes according to their roles in the generative process: white represents latent variables, gray represents observable variables, rectangles indicate that the variables require loop computation, and arrows represent parameters and the directions of variables.

**Fig 3:**
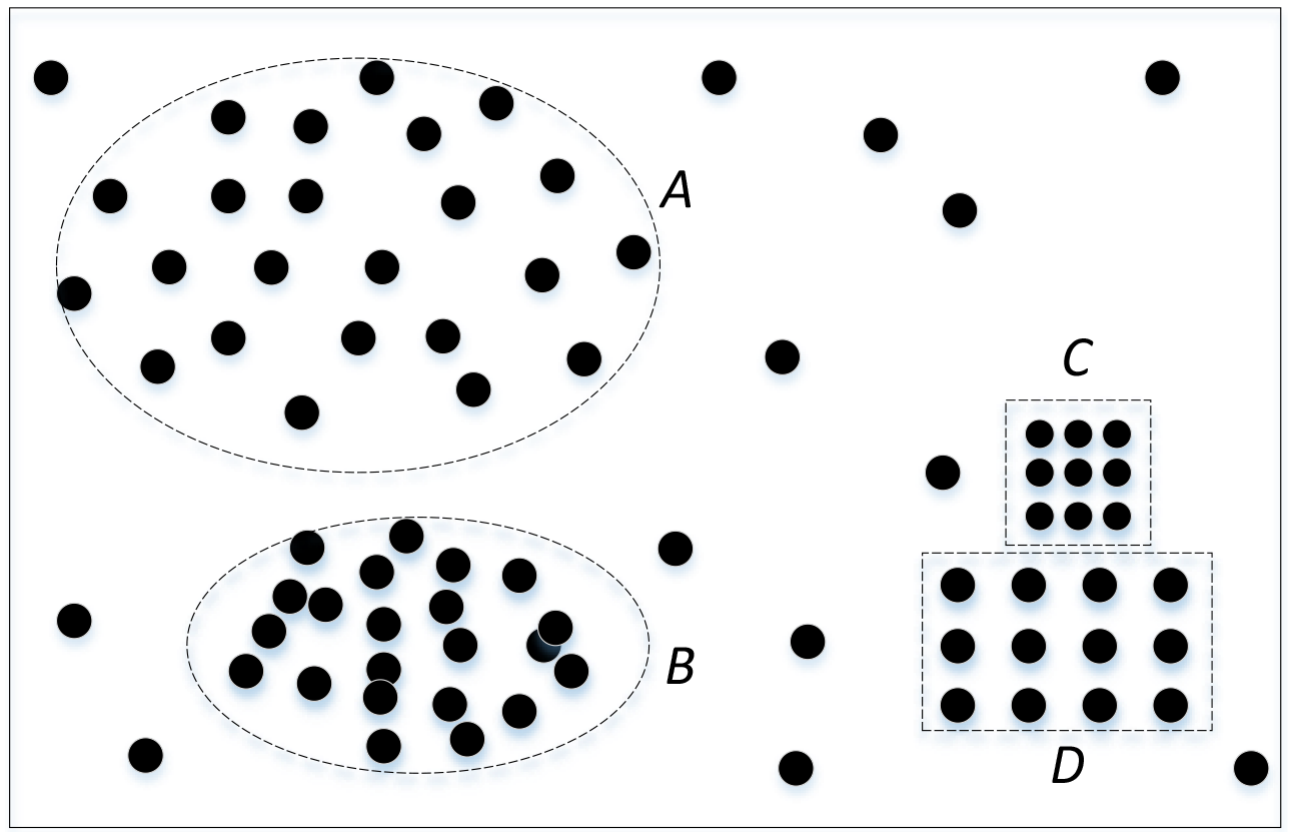
Space clusters of different density. This figure shows four different density clusters to be identified by the OPTICS method.

In Fig 3, α and η represent the input parameter of the distribution of the Dirichlet region functional zone and the distribution of the residential travel functional zone, respectively. Assuming that a certain region has K functions, β is a matrix of K*M (M represents the number of trips of all residents in city D). Each β_*k*_ is a distribution throughout the city. The proportion of a functional zone in the dth region is *θ*_d_, and *θ*_d,k_ is the proportion of functional zone K in city d. The functional zone distribution of city d is Z_d_, and Z_d,n_ represents the functional distribution of resident n in city d. The number of observed trips of residents in city d is represented by W_d_, and W_d,n_ stands for the number of trips of resident n in city d.

Moreover, the α value from the DMR model accounts for the POI feature vector of each region *α*_*i*_. For example,$\alpha_{i,k}=exp(X_{i}^{T}\lambda_{k})$. Therefore, different combinations of POI category distributions will yield distinct α values. Hence, the activity distribution is the sum of the POI features and mobility patterns. Finally, by applying DMR and inputting the mobility patterns and POI features, we obtain the activity distribution of each region and the mobility pattern distribution of each activity.

Mobility pattern M is recorded as a tuple:

$$M=<\;O_{i},T_{i},D_{j},T_{j}>$$ (1)

where *O*_*i*_, *T*_*i*_, *D*_*j*_, and *T*_*j*_ represent the departure zone, departure time from the departure zone, destination zone, and arrival time in the target zone, respectively. If one departs from zone S at time T and arrives in destination zone X, then “O_X_T” is the mobility pattern of the departure zone, where “O” stands for the departure zone S, and “S_D_T” is the mobility pattern of the destination zone, where “D” represents the destination zone X.

First, we extract the origin-destination (O/D) pairs from the massive floating car data set and link the O/D pair with the research area to determine the area of each trip. Then, Formula 1 is used to perform one-to-one mapping from the O/D pairs to the mobility pattern. An example of mobility pattern is 305_D_21, which represents a taxi trip starting from area 305 and ending in area 21. Next, we build a DMR model based on the Mallet platform [59] to process the mobility patterns. After processing the DMR model, we obtain the probability distribution of each topic of each functional region and employ ordering points to identify clustering structure (OPTICS) to determine the commercial zone. Finally, we identify and analyze the commercial zone.

The OPTICS method is a density-based spatial clustering method. This method overcomes the limitation of global parameters set by the density-based spatial clustering of applications with the noise (DBSCAN) algorithm, which cannot perform clustering operations under non-uniform spatial data distributions. The OPTICS method is an improved DBSCAN algorithm. To illustrate, Fig 3 shows four different density clusters, A, B, C and D; if the value of Ɛ is large, the A cluster can be identified, but neither A and B nor C and D can be separated. Furthermore, when the value of Ɛ is small, cluster A will be difficult to recognize.

To solve the problem of spatial data density distribution, the OPTICS algorithm is added to the density-based clustering sorting.

Here, sort represents the density structure of spatial data, and the information expressed is equivalent to the spatial clustering structure obtained with different parameter values (e.g., neighborhood radius). Unlike the DBSCAN algorithm, the OPTICS algorithm stores the order in which spatial entities are processed, providing a series of neighborhood radius parameter values and allowing spatial entities to be clustered from high density to low density at one time. To achieve this operation, the OPTICS algorithm introduces the concepts of core distance and reachable distance, calculating the kernel distance and a suitable reachable distance for each spatial entity. Using this information, we can identify several clusters of data with different densities.

## Results

**Fig 4:**
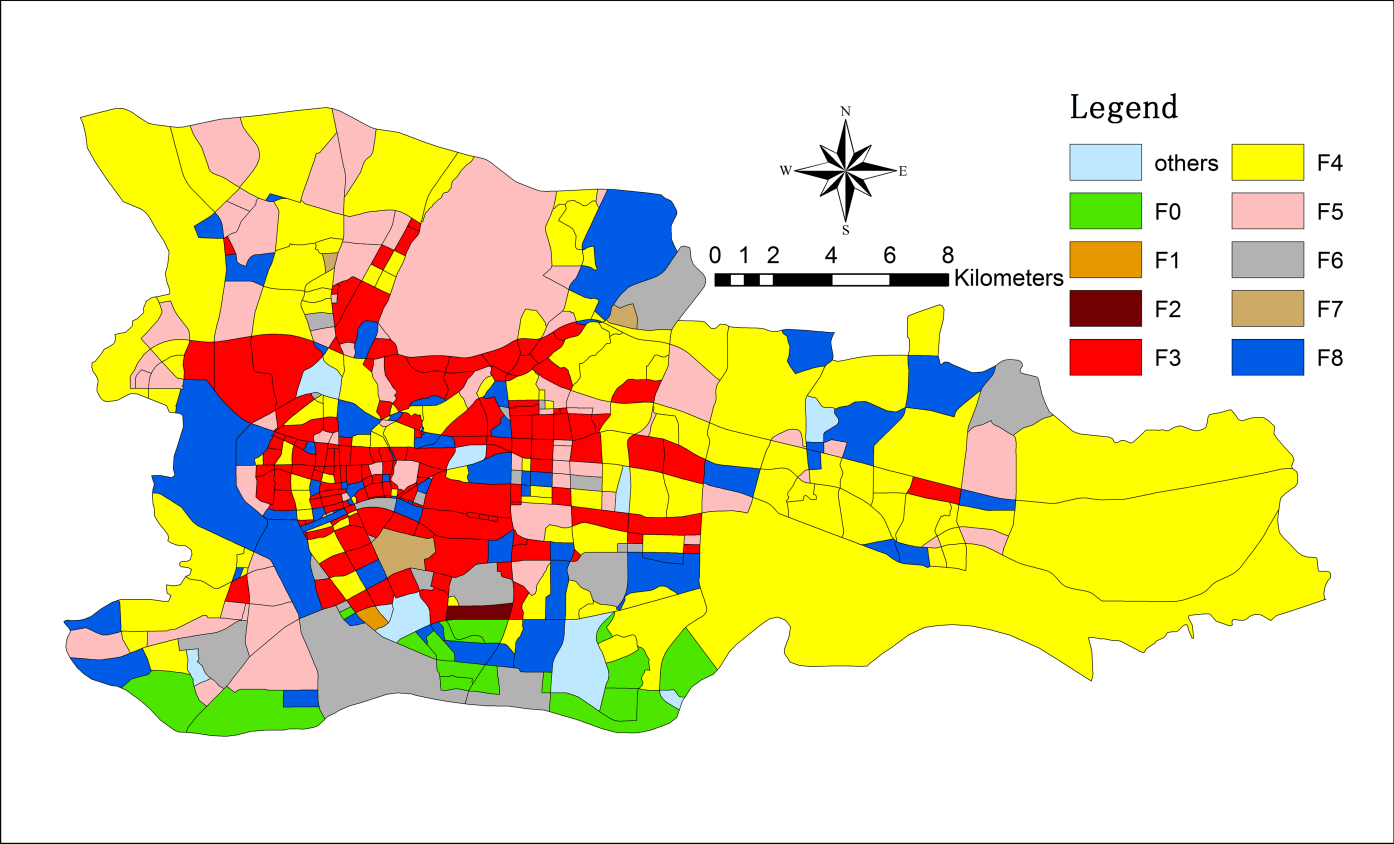
The functional zones in Guangzhou in 2009.

**Fig 5:**
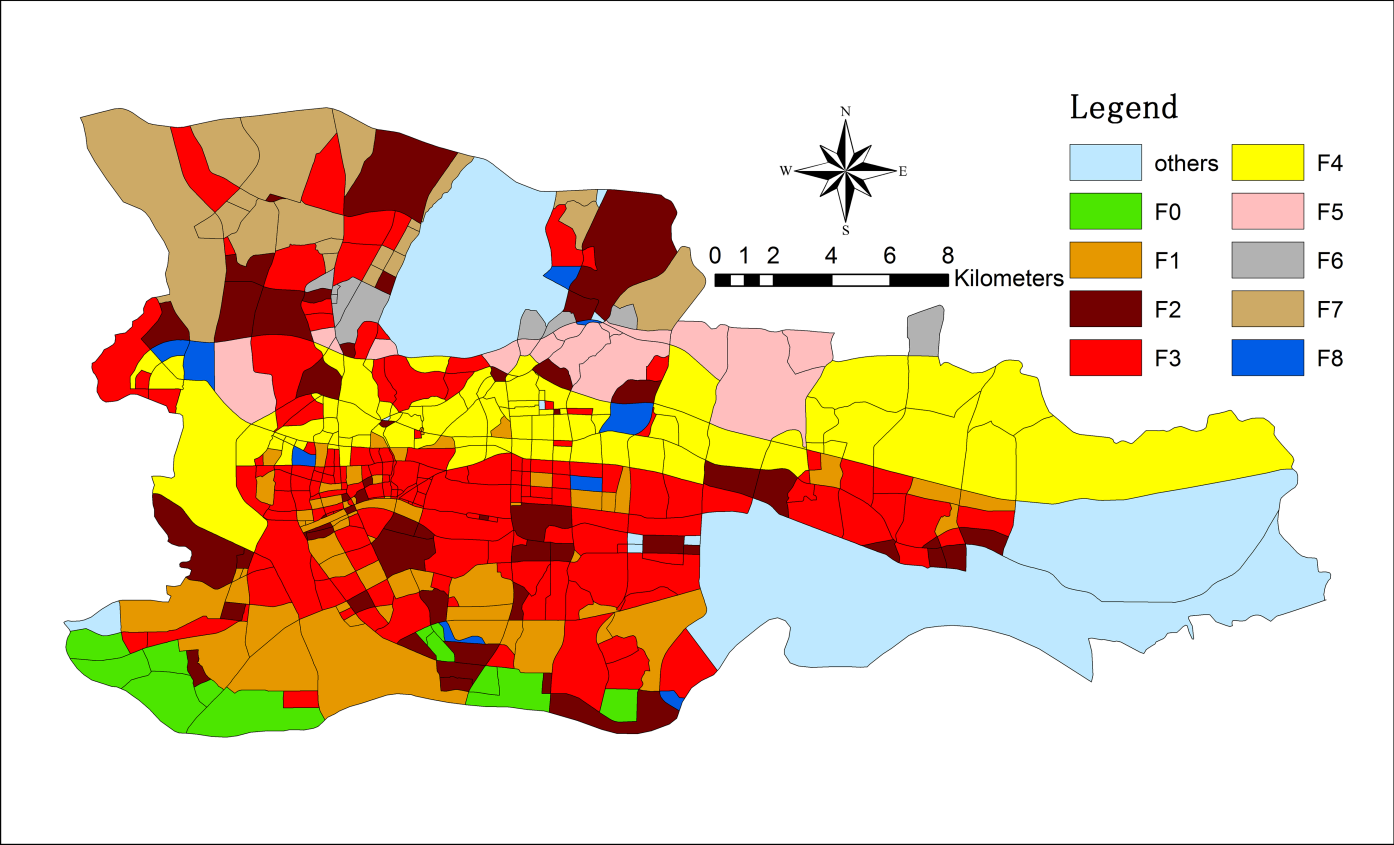
The functional zones in Guangzhou in 2013.

Following the abovementioned procedures and OPTICS clustering, we obtain the resulting commercial structure of Guangzhou city (as displayed in Figs 4 and 5).

### Discovering commercial space

After constructing a model to determine the zone clustering of areas with the same functions, it is essential to identify functional zones based on their actual functions. There are various criteria for urban functional zoning, and the partitioning results are distinct. Identification of all of the functional areas was performed in a previous paper [34] and is not repeated here.

This paper partitions the study region according to social functions, residents’ needs, and other factors to identify the commercial zone. In our opinion, a mature commercial zone requires a long-term development strategy that provides a balanced combination of shopping, restaurants, activities, facilities, and other attractions near the area. The travel pattern not only includes travel peaks in the morning and evening but also appeals to the city’s residents. Therefore, we calculate the distribution of POIs in the research area and the flow of taxis to the commercial zone each week, day, and hour to identify the commercial structure. Because the taxi flow in 2009 is similar to that in 2013, we use only the taxi flow information from 2013 in further analyses due to the manuscript length limitations.

**Fig 6:**
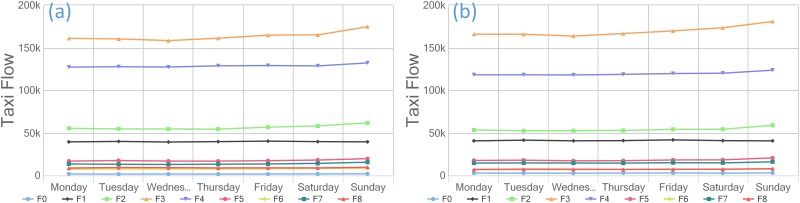
The different functional zones of the (a) departures and (b) arrivals of taxis in 2013.

**Fig 7:**
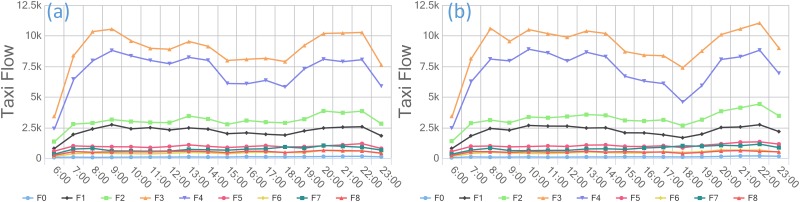
(a) Departure flows in different zones on weekdays (b) Departure flows in different zones on weekends.

As displayed in Figs 4 and 5, we obtain the spatial distribution of nine zones (F0~F8) through clustering. Figs 6 and 7 demonstrate the travel flows on weekdays and weekends in different zones, i.e., the temporal distributions of departures and arrivals. Zone F3 maintains the highest flow. Moreover, the flow in this zone increases significantly on weekends, which implies that there are significantly more visits to this zone on weekends.

**Fig 8:**
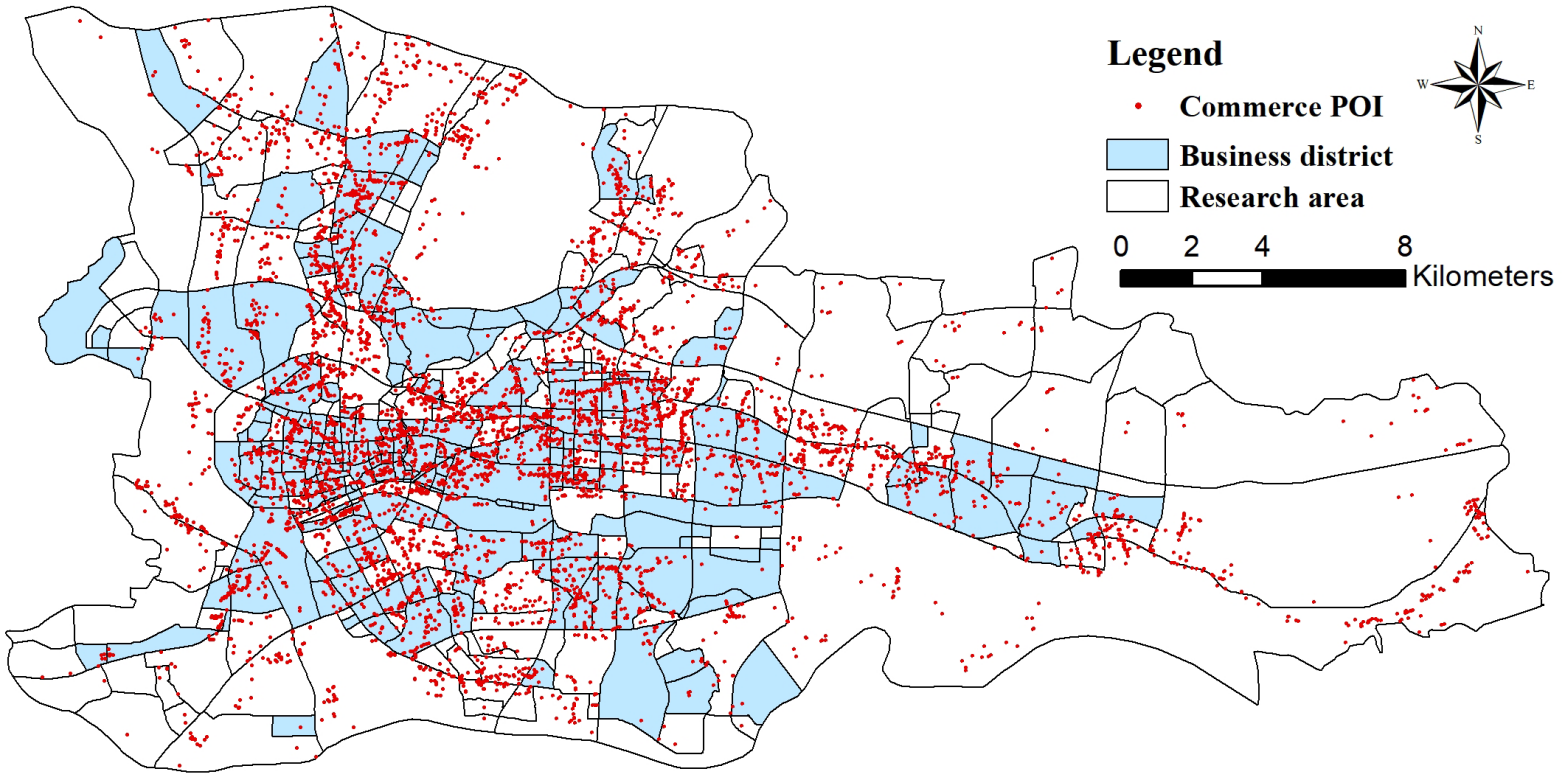
The distribution of commercial points of interest. The figure shows the business districts from Fig 5 and commercial POIs in the research area.

In addition, we select POIs for banking, insurance, restaurants, accommodations, recreation, and other services as the distribution types in the commercial zone and obtain the results shown in Fig 8.

By further analyzing the departure flows on weekdays/weekends, we find that the flows in zones F3 and F4 are essentially identical. Nevertheless, the overall flow in zone F4 is smaller than that in zone F3 but undergoes more changes. Meanwhile, based on the distribution types of POIs, zone F3 has the widest coverage of different types of POIs, among which restaurants, shopping, and offices account for relatively large proportions and are associated with integrated service attributes. In contrast, zone F4 features residential buildings and living services associated with living space attributes. Therefore, we believe that zone F3 is a commercial zone, whereas zone F4 is a mature residential zone.

In addition, zones F3 and F4 have similar “pendulum traffic” patterns because the commercial areas and residential areas in Guangzhou are highly mixed. Because the commercial zone is more attractive than the residential zone, it has a larger flow of people. In terms of the flow variations, the residential zone displays more regularity in travel behaviors in the morning and evening, resulting in more variations in the flow. However, the commercial zone attracts both regular residents and irregular visitors and thus has a flow that slightly varies. This variation constitutes the major criterion for us to distinguish between the commercial zone and the mature residential zone.

### Human mobility based on taxi data in commercial regions/zones

**Fig 9:**
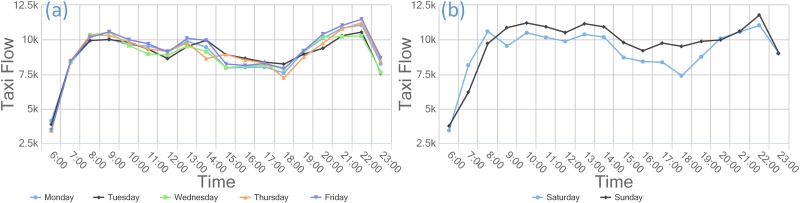
Departure flows in the commercial zone on (a) weekdays and (b) weekends in 2013.

**Fig 10:**
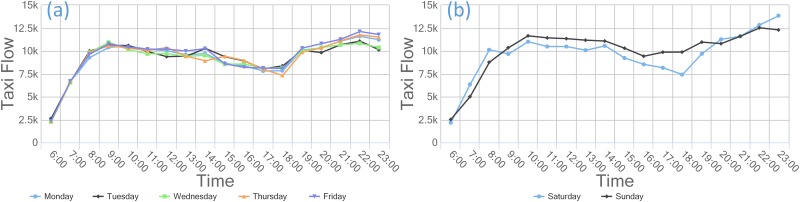
Arrival flows in the commercial zone on (a) weekdays and (b) weekends in 2013.

By examining the times of flows in the commercial zone based on the departure and arrival flow data, we find that the flow in this zone displays well-defined regularity on weekdays and weekends. As shown in Figs 9 and 10, the departure and arrival flows in the commercial zone reach peaks in the morning and evening on weekdays. The arrival flows peak at 9:00–10:00 am, reflecting the need of commuters to go to work during that period. The evening flow begins to increase at 7:00 pm, and its peak flow surpasses that in the morning, indicating that many residents outside the functional zone also travel to the commercial zone for work, shopping, recreation, etc. In addition, although the weekend flow is higher than that on weekdays, it does not change significantly over time. Thus, a more balanced distribution of flows in the commercial zone is observed on weekends, which conforms to the typical mobility pattern in the commercial zone. Moreover, there is a distinct growth in the flow of arrivals to the commercial zone in the early hours of Saturday, which can be explained by the unique weekend nightlife in Guangzhou, a first-tier city.

### The evolution of the urban commercial structure after the financial crisis and result verification

**Fig 11:**
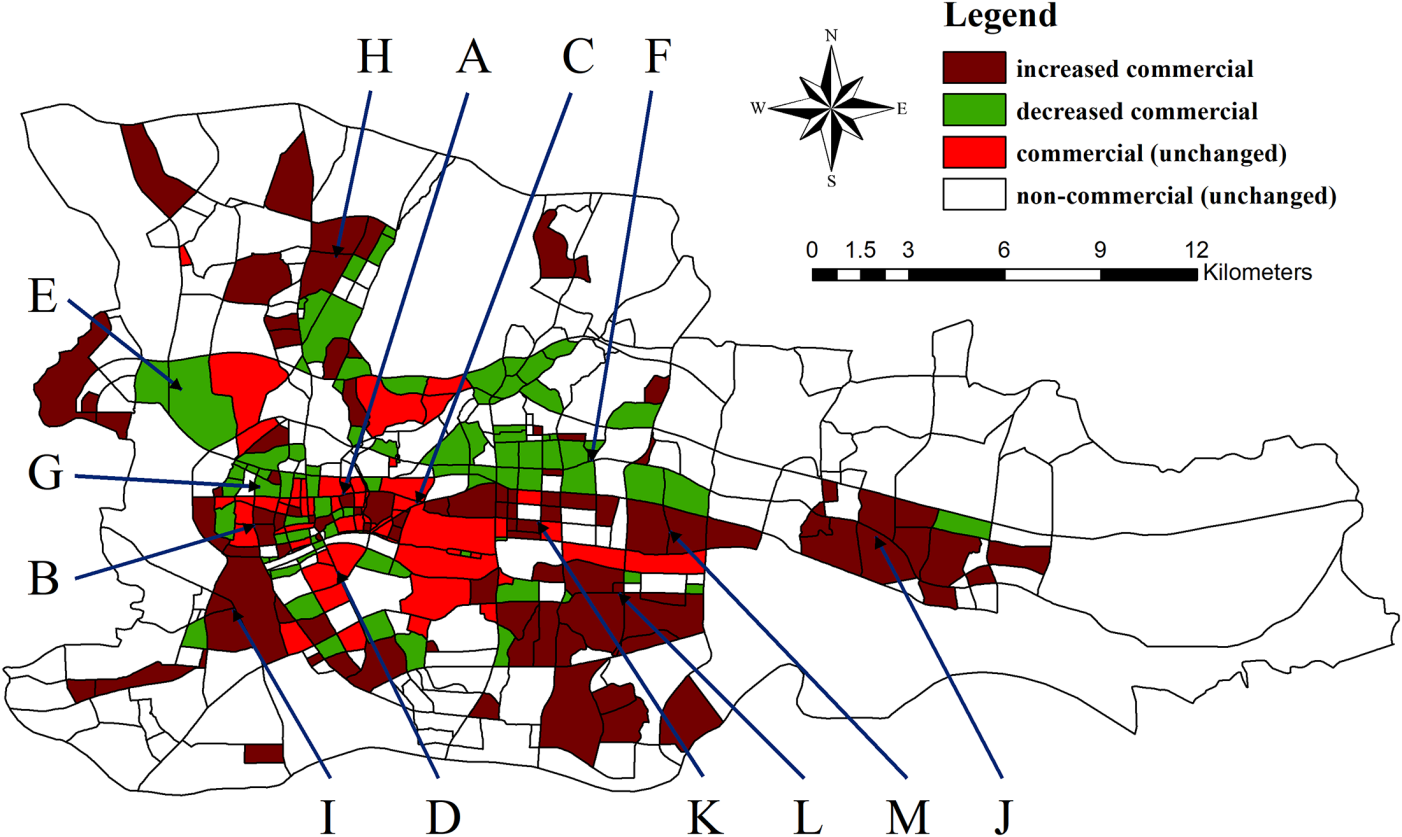
Evolution of the commercial structure from 2009 to 2013. The figure shows the increased commercial, decreased commercial, unchanged commercial and non-commercial districts in the study area from 2009 to 2013.

The 2008 global financial crisis caused considerable changes to the development of many countries and cities, and Guangzhou is no exception. Using taxi trajectory data, we can analyze some changes to the commercial structure of Guangzhou, which are shown in Figs 4 and 5. The results reveal that the commercial space in 2009 was distributed in fragments around the city center. By 2013, the number of business fragments decreased, and many fragments combined and spread, covering the entire city of Guangzhou (as displayed in Fig 11). This result was likely caused by the enhancement in residents’ quality of life and the diverse economic development led by the service industry, which promoted the change in Guangzhou’s commercial structure from a single center to a polycentric network.

In different regions of a city, commercial spatial structures undergo different changes: some commercial zones remain, some vanish, and some expand. Located on the central axis of old town, the two famous business circles, Beijing Road (A) and Shangxiajiu Pedestrian Street (B), have time-honored cultures and business traditions as well as a variety of stores (as shown in Fig 11). They are retail business circles with integrated services. From 2009 to 2013, their coverage area increased slightly. Thus, the two business circles have sprawled outward and developed into new business zones. As two important community business circles, China Plaza (Nonglinxia Road) (C) and Jiangnanxi (D) serve primarily the surrounding local residents. With a static and limited range of target customers, these two business circles did not experience significant changes in their spheres of influence from 2009 to 2013. Fig 11 shows the distribution of the remaining business zones in the region.

Some business zones have lost their original business attributes due to upgrades and transformations. The Xiwan Road-Zengcha Road area (E), which has gradually developed into a hub of wholesale markets and logistic and freight services, is an example. Conversely, some areas experienced increases in residential or cultural and recreational functions and gradually converted to living spaces from commercial spaces during the period 2009–2013. Shipaidong Road-Wushan Road (F) and the northern part of Xianliedong Road to Yanling Road are examples of increased residential function. For example, the Gangding and Shipai area has numerous residential buildings but lacks sufficient offices and warehouses. Additionally, its main industry, IT retail sales, has suffered in recent years due to the economic downturn and the impact of electronic commerce, which have greatly decreased its commercial influence. Examples of growth associated with cultural and recreational functions include the northern part of Zhongshanqi Road to Zhongshanba Road (G) and the areas surrounding Tianhe Park. For example, the Chen Clan Ancestral Hall located on Zhongshanba Road has become a tourism landmark in Guangzhou.

The new business zones reflect the long-term strategic plan in Guangzhou. The city’s commercial spatial structure has changed from a cluster in the city center to a network covering the entire city and its surroundings. The area of Guangbai and Qifu Road (H) around the new town possesses integrated commercial functions and promotes the development of the new urban district in the northern part of the city. Due to the prime location of Fangcun, the west bank of the Pearl River has developed a commercial circle, Huadiwan-Fangcun (I), which connects and reaches to neighboring cities. The development of Huangpu District (J) in the eastern part of the city has proven effective, and new business zones are gradually forming there. Numerous emerging business districts are developing rapidly and are connected to the existing business areas along the central axis, including the high-end office zone of Zhujiang New Town (K) and exhibition centers such as the International Convention and Exhibition Center in Pazhou (L). For instance, Yuancun, an extension of the Zhujiang New Town CBD, was transformed from an old industrial zone into a residential area and then into a financial hub with financial and commercial functions from 2009 to 2013. Thus, it is currently associated with a commercial structure. In summary, the developments of these emerging commercial zones are based on the planning strategy in Guangzhou: “develop the south, optimize the north, move toward the east, and connect the west”. The city is adjusting its spatial structure to enhance its city functions and to convert from a single center to a polycentric network.

We use data from the “Guangzhou Large-scale Retail Business Network Development Plan (2011–2020)” released by the Guangzhou Planning Bureau in 2013 as the standard to verify the commercial functional zoning results produced in our research. The data show that by the end of 2010, Guangzhou completed the construction of commercial functional areas in five metropolitan zones (Beijing Road, Central City East, Agriculture and Forestry Road, Zhongshan Three Road, and Thirteen Line-Shangxiajiu Tianhe Road) and seven district zones (Jiangnan, Xiaogang, Shijing-Xinshi, Dashadi, Shiqiao, Licheng, and Jiekou). Excluding Shiqiao, Licheng, and Jiekou, which are shown in Figs 4 and 5, these commercial functional areas are outside the study area. Thus, the proposed method can effectively identify the distribution of business districts.

## Discussion

**Fig 12:**
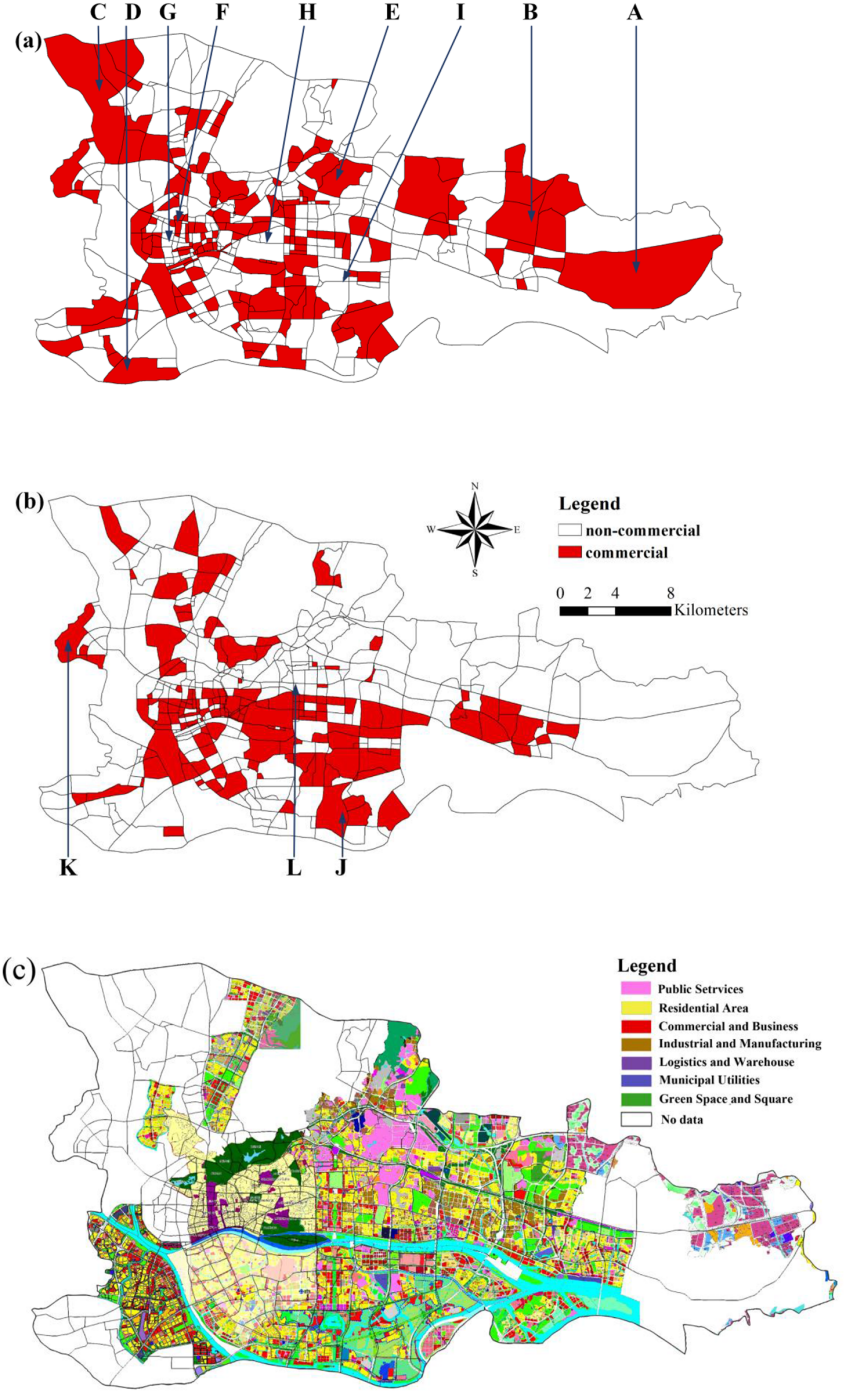
(a) Commercial zone results based on POI data, (b) commercial zone results based on GPS and POI data, and (c) land-use map. These figures show three commercial districts in the research area. (a) presents the commercial zone results based on the POI data.

To validate the results regarding the commercial zones, this research uses POI data to identify commercial areas. The steps are as follows: First, the POI data are divided into 29 types. Second, the probability of the occurrence of each type of POI in each research area is calculated. Third, the statistical results are used as the input data in the DMR model. Finally, the OPTICS clustering method is used to cluster the results of the DMR model and obtain the results for the commercial zones (as displayed in Fig 12). The results based on the POI data and the DMR model are shown in Fig 12(a) and 12(b) presents the commercial zones based on the GPS data, POI data and DMR model.

Land-use maps are very difficult to obtain in China. Therefore, through other related projects, this study obtains a limited land use status map of the research area, which is used for comparison with the results shown in Fig 12(a) and 12(b). Although this land-use map is limited, it is very informative. Through comparative analysis, this study finds that POI data and DMR models can be used to correctly identify some commercial zones. However, many areas are misidentified as commercial areas, especially in remote areas, such as the mountainous area (A) and the mountainous plains mixed area (B) in the east of Huangpu District, the agricultural area (C) around the Beijing-Shenzhen line, and the southwest corner of the ring expressway (D). These areas include some commercial facilities, and the POI data for these areas are very sparse. Therefore, POI-sparse areas with few commercial POI facilities can be misidentified as commercial areas. With the development of the city, although many commercial facilities are preserved, some commercial areas gradually transform into residential areas, such as Wushan Campus (E) and Liuhuahu Park (F). Furthermore, the areas of Shangxiujiu (G), Yuexiu District (H) and Chigang (I) belong to traditional commercial areas but are not identified by the DMR model, which may be caused by the inability of POI data to capture the long-term development of these areas.

Wu used POI data to identify multiple types of commercial centers [60]. This author’s results showed that the commercial area structure presents a dual-core distribution, with the urban services center and public service center located in Yuexiu District and the business center and financial center located in Tianhe District. These results are consistent with our findings shown in Fig 12(b). However, this research also revealed that POI data have the characteristics of large data volume but low information, as they ignore detailed information on the development characteristics and building age of geographical entities. Therefore, when identifying the boundary of the central area through POI data, the development characteristics and level information of the central area are easily overlooked, which will affect the accuracy of the boundary. Simultaneously, the model results cannot be used to analyze the development status of the central area.

Overall, the extraction of commercial areas using POI data can reflect the distribution of commercial facilities to some extent. However, the classification results cannot reflect the distribution of actual commercial zones. It is difficult to differentiate commercial areas based only on POI data; on the one hand, there are always more commercial POIs than residential POIs in an area at the same time. Therefore, for areas in which mining distribution features, most regions generally have commercial properties. On the other hand, due to the limitation of POI data, the model tends to ignore some key information such that the frequency of POIs cannot represent the potential structure of each region well.

This study also compares the results of Fig 12(b) with the land-use map in Fig 12(c). Although there are some misclassifications in the results, such as around the Tuhua overpass (J) and Shabei overpass (K) (in the northwestern and southeastern corners of the city ring), some emerging communities have increased in population but lack the support of commercial facilities, which are insufficient to form a new commercial space. Furthermore, in the traditional business district of Sports Center (L), the large number of people in transit reflects the high degree of functional mix, which makes the DMR model unable to identify the region as a commercial space. Overall, the results of the DMR model are closer to the actual land-use conditions than are the results of the DMR model, indicating that the use of GPS and POI data to discover urban functional zones provides more reliable results than the DMR model.

## Conclusion

From the traditional perspective, qualitative analyses of urban commercial spatial structures and the associated changes have certain limitations, and the temporal resolution is low. With big data growing in popularity, obtaining information regarding the changes in a commercial spatial structure in a timely and effective manner has become possible. Based on the concepts of text categorization and traditional urban functional zoning, this paper uses big data associated with the daily movements of people and a semantic theme model (DMR model) to study the urban commercial districts in Guangzhou. Based on traditional methods, the study explores the research concepts and provides a new method that uses residents’ behaviors to study the urban commercial spatial structure; the results are important for guiding future studies and management strategies.

First, this paper discusses a new method of identifying urban commercial space, and the results are in accordance with the urban planning strategy in Guangzhou. This result suggests that taxi and POI data and the DMR model can be used to determine the spatial distribution and changes in an urban commercial space. In addition, after obtaining the distribution of commercial districts Guangzhou in 2009 and 2013, the paper analyzes the trends and factors related to the evolution of the commercial space. The study also shows that the urban commercial structure in Guangzhou gradually changed from a single-center model to a multi-center model with dispersed clusters from 2009 to 2013. Moreover, the distribution of the other functional districts in the city and the overall spatial structure changed. Although the distribution remains heterogeneous among regions, these regional differences will likely diminish.

Furthermore, through the analysis of different regions, we find that the evolution of urban commercial districts reflects not only temporal changes but also specific geographical features. Early or historical commercial circles and community-oriented commercial districts for residents were largely retained. The commercial development of historic commercial circles was accompanied by a revival in the tourism industry. Additionally, community-oriented commercial districts are less likely to change because of the stability of the service groups in those areas. Moreover, under certain conditions, the commercial structure will begin to "recede" or "improve" based on a multi-center model, although the reasons for the two trends are different. Recession occurs because other functions in the region (such as leisure and residential functions) gradually become dominant, as they are affected by market and consumer behaviors. Conversely, improvement is associated with the macro-control and guidance provided by city planning, and these advantages are used to overcome the limitations of market-based economic development.

Although the study identifies and analyzes the commercial space in Guangzhou from the aspect of a floating car movement mode, the distributions of commercial space in different cities are heterogeneous, and the evolution processes are different. The factors that affect changes in the distribution of commercial space in different regions are complicated and include the economy, society, culture, and other factors. If we rely only on the floating car movement mode, we cannot obtain a comprehensive summary of the movement in a city; however, the innovative research methods applied and the resulting trends are still noteworthy. Therefore, we use the multi-source traffic model based on economic, social, cultural, and other factors to study the urban commercial space structure and changes in China, and this model will be used and improved in future studies.

## Supporting information

S1 Table Data used in Fig 6. This comma separated value (CSV) file contains the departure taxi flow presented in Fig 6(a) and the arrival taxi flow data presented in Fig 6(b). The first column contains functional zone data, and the other column contains the daily taxi flow of each functional zone.
(CSV)

S2 Table Data used in Fig 7. This comma separated value (CSV) file has 9 columns; the first column contains time data, and the remaining columns provide flow data for each functional zone. The data used in Fig 7(a) are the departure flows on weekdays, and those used in Fig 7(b) are the departure flows on weekends.
(CSV)

S3 Table Data used in Fig 9. This comma separated value (CSV) file has 8 columns; the first column contains time data, and the remaining columns provide daily departure flows in the commercial zone.
(CSV)

S4 Table Data used in Fig 10. This comma separated value (CSV) file has 8 columns; the first column contains time data, and the remaining columns provide daily arrival flows in the commercial zone.
(CSV)
